# Room-Temperature-Processed Amorphous Sn-In-O Electron Transport Layer for Perovskite Solar Cells

**DOI:** 10.3390/ma13010032

**Published:** 2019-12-19

**Authors:** Seungtae Baek, Jeong Woo Han, Devthade Vidyasagar, Hanbyeol Cho, Hwi-Heon HA, Dong Hoe Kim, Young-Woo Heo, Sangwook Lee

**Affiliations:** 1 School of Materials Science and Engineering, Kyungpook National University, Daegu 41566, Korea; en5840@knu.ac.kr (S.B.); wjddn0820@knu.ac.kr (J.W.H.); jhb1005@gmail.com (H.C.);; 2 Department of Nanotechnology and Advanced Materials Engineering, Sejong University, Gwangjin-gu, Seoul 05006, Korea; donghoe.k@sejong.ac.kr

**Keywords:** tin-indium-oxide, room temperature, perovskite solar cell, electron transport layer, band structure, electrical property

## Abstract

We report amorphous tin-indium-oxide (TIO, Sn fraction: >50 atomic percentage (at%)) thin films as a new electron transport layer (ETL) of perovskite solar cells (PSCs). TIO thin films with Sn fraction of 52, 77, 83, 92, and 100 at% were grown on crystalline indium-tin-oxide (ITO, Sn fraction: ~10 at%) thin films, a common transparent conducting oxide, by co-sputtering In_2_O_3_ and SnO_2_ at room temperature. The energy band structures of the amorphous TIO thin films were determined from the optical absorbance and the ultraviolet photoelectron spectra. All the examined compositions are characterized by a conduction band edge lying between that of ITO and that of perovskite (here, methylammonium lead triiodide), indicating that TIO is a potentially viable ETL of PSCs. The photovoltaic characteristics of the TIO-based PSCs were evaluated. Owing mainly to the highest fill factor and open circuit voltage, the optimal power conversion efficiency was obtained for the 77 at%-Sn TIO ETL with TiCl_4_ treatment. The fill factor and the open circuit voltage changes with varying the Sn fraction, despite similar conduction band edges. We attribute these differences to the considerable changes in the electrical resistivity of the TIO ETL. This would have a significant effect on the shunt and/or the series resistances. The TIO ETL can be continuously grown on an ITO TCO in a chamber, as ITO and TIO are composed of identical elements, which would help to reduce production time and costs.

## 1. Introduction

Organic-inorganic hybrid perovskites are characterized by high absorption coefficients [1], long carrier diffusion lengths [2,3], easily tunable band gaps [4,5], low-temperature solution processes, and low costs [6,7,8]. Based on such characteristics, these perovskites have attracted considerable attention as light absorbing materials of perovskite solar cells (PSCs) [9,10]. A PSC employs one of two structural configurations, i.e., electrode/hole transport layer (HTL)/perovskite/electron transport layer (ETL)/transparent conductive oxide (TCO) or electrode/ETL/perovskite/HTL/TCO [11,12,13], referred to as regular (n-i-p) and inverted (p-i-n) type PSCs, respectively. Depending on the shape of the ETL, a PSC is classified as either a mesoscopic or a planar structure. In the former, the charge transport layer on TCO is composed of a nanoparticle-based mesoporous film, whereas in the latter, each charge transport layer is a compact thin film [11,12,14,15].

ETL plays an important role in extracting the excited electrons (or blocking the holes) from the light absorption layer (LAL) and transporting these electrons from the LAL to the electrode. The conditions required for an ETL of a PSC are summarized as follows: (i) appropriate conduction band minimum (CBM) and valence band maximum (VBM)-the CBM between those of the LAL and the TCO, to inject the electrons from the LAL to the TCO, and the VBM lower than that of the LAL to block the holes [16,17]; (ii) a high electron mobility and a moderate carrier density, to promote efficient transport of electrons [16,18]; (iii) a wide band-gap, to transmit the incident light associated with regular-type devices [16]; (iv) long-term stabilities against heat, oxygen, and humidity [19,20,21,22,23,24]. From these aspects crystalline metal oxides, such as TiO_2_ [25,26,27], SnO_2_ [28], BaSnO_3_ [29], Nb_2_O_5_ [30], WO_x_ [31,32], and Zn_2_SnO_4_ [33], have been studied for use as the ETLs of PSCs.

However, to realize good electrical properties of such crystalline oxides, the oxides need in most cases a post-heat treatment [34,35,36,37]. This treatment is undesirable, owing to the additional production time and costs as well as the difficulty of realizing flexible devices, because polymer-based flexible substrates deteriorate at high temperatures. In addition, a crystallized film has a rough surface due to the formation of sharp-edged facets, grains, and grain boundaries, which trigger cracking during bending cycles [35,38,39]. Alternatively, it has been reported recently that amorphous oxide-based ETLs can be grown via low-temperature processes, such as solution spin-coating, chemical bath deposition, and vapor deposition [38,39,40]. Moreover, amorphous films exhibit greater bending durability than crystalline films [35,38,39].

In this study, we report a tin-indium-oxide (TIO; Sn fraction: >50 atomic percent (at%)) amorphous thin film as a new ETL of PSCs. Although ITO has been widely used as a TCO, its use as an ETL remains unexplored. TIO amorphous thin films were grown on ITO TCO by co-sputtering SnO_2_ and ITO at room temperature. The crystallinity, optical properties, and electrical properties of the films are investigated as a function of the Sn fraction. The energy band structures of the amorphous TIO thin films are determined from optical absorbance spectra and ultraviolet photoelectron spectra. Moreover, TIO-ETL-based PSCs are demonstrated with a methylammonium lead triiodide (MAPbI_3_) perovskite light absorber. 

## 2. Materials and Methods

### 2.1. Materials

Methylammonium iodide (MAI, ≥99.5%, Polymer Light Technology Corp., Xi’an, China), Lead(II) iodide (PbI_2_, 99.9985%, Tokyo Chemical Industry Co., Nihonbashi-honcho, Chuo-ku, Tokyo, Japan), Lead(II) bromide (PbBr2, ≥98.0%, Tokyo Chemical Industry Co., Nihonbashi-honcho, Chuo-ku, Tokyo, Japan), Formamidinium iodide (FAI, ≥99.0%, Greatcell Solar, Queanbeyan, New South Wales, Australia), Methylammonium bromide (MABr, ≥99.0%, Greatcell Solar, Queanbeyan, New South Wales, Australia), Cesium iodide (CsI, 99.9%, Sigma-Aldrich, St. Louis, MO, USA) Dimethyl sulfoxide (DMSO, ≥99.9%, Sigma-Aldrich, St. Louis, MO, USA), N,N-Dimethylformamide (DMF, 99.8%, Sigma-Aldrich, St. Louis, MO, USA), Diethyl ether (DEE, ≥99.7%, Sigma-Aldrich, St. Louis, MO, USA) were used for preparing the perovskite thin films. Spiro-MeOTAD (≥99.9978%, Luminescence Technology Corp., New Taipei City, Taiwan, China), FK 209 Co(III) TFSI salt (Cobalt compound, ≥98.0%, Luminescence Technology Corp., New Taipei City, Taiwan, China), Lithium bis(trifluoromethanesulfonimide) (Li-salt, 99.0%, Acros Organics, Geel, Belgium), 4-tert-Butylpyridine (tBP, 96%, Sigma-Aldrich, St. Louis, Mo, USA), Acetonitrile (99.8%, Sigma-Aldrich, St. Louis, MO, USA), and Chlorobenzene (CB, 99.8%, Sigma-Aldrich, Haverhill, MA, USA) were used for making HTL thin films. Tin(IV) oxide (SnO_2_ col, 15% in H_2_O colloidal dispersion, Alfa Aesar, Haverhill, MA, USA) was used for preparing SnO_2_ thin films. Titanium tetrachloride (TiCl_4_, ≥99.0%, Junsei Chemical Co., Nihonbashi-honcho, chou-ku, Tokyo) was used for the TiCl_4_ surface treatment.

### 2.2. Sn-In-O (TIO) Thin Film Deposition

TIO amorphous thin films were deposited by means of co-sputtering In_2_O_3_ and SnO_2_ targets using an RF magnetron sputtering system at room temperature. The substrates used were ITO (Samsung Corning^®^, on Eagle glass, 6–7 Ω/sq), slide glass, and sapphire wafer depending on the analysis performed. Each substrate was cleaned sequentially with acetone, deionized water, and isopropanol in an ultrasonic bath twice for 3 min each. The substrates were further cleaned for 20 min by means of a UV-Ozone (UVO) surface treatment, prior to the deposition of the TIO thin films. The composition of the films was controlled by adjusting (i) the distance between the targets and the substrates, and (ii) the gun powers of the In_2_O_3_ target (25 W to 75 W) and the SnO_2_ target (75 W to 200 W). A mixture gas (volume ratio of O_2_ to Ar = 0.001) at a flow rate of 100 sccm was used for the process gas.

### 2.3. Device Fabrication

Organic-inorganic hybrid perovskite layers were coated on the TIO ETL. Size of the substrates were 2 cm × 2 cm. In some cases, the substrates were subjected to a TiCl_4_-treatment prior to the perovskite coating process. During this treatment, the films were immersed in an aqueous TiCl_4_ solution (0.05 M) at 70 °C for 30 min, and then rinsed with deionized water and isopropyl alcohol. All substrates were UVO treated for 20 min immediately prior to the deposition of the perovskite layer coating. A perovskite precursor solution with MAPbI_3_ was prepared by dissolving MAI:PbI_2_:DMSO at a molar ratio of 1:1:1 at 52 wt.% in DMF. The precursor was dropped onto the UVO-treated TIO film and spin-coated at 4000 rpm for 20 s. During the spinning, 0.5 mL of DEE was dropped. After the coating process, the samples were annealed at 65 °C for 1 min, and then at 130 °C for 10 min. Stability tests for the ITO-ETL-based PSCs were performed using a tri-cation perovskite. For this, a perovskite precursor solution with Cs_0.05_(FA_0.87_MA_0.13_)_0.95_Pb(Br_0.13_I_0.87_)_3_ was prepared by dissolving CsI, FAI, MABr, PbI_2_, and PbBr_2_ in DMF: DMSO (v/v) = 4:1 at a concentration of 1.4 M with the composition [41]. The precursor was dropped onto the UVO-treated In-Sn-O thin film and spin-coated at 5000 rpm for 30 s. During the spinning, 0.2 mL of CB was dropped. After the spin coating, the samples were annealed at 150 °C for 10 min. The HTL was coated on the perovskite layer by spin-coating 20 µL of spiro-MeOTAD mixed solution at 3000 rpm for 30 s. This mixed solution was prepared by sequentially adding 18 μL of Li-TFSI solution and 14.7 mg of Co-TFSI to 1 mL of spiro-MeOTAD solution. The Li-TFSI solution was prepared by dissolving 566 mg of Li-TFSI in 1 mL of acetonitrile. Furthermore, the spiro-MeOTAD solution was prepared by sequentially dissolving 80 mg of spiro-MeOTAD and 32 μL of 4-tert-butylpyridine in 1 mL of chlorobenzene. Afterward, the Au electrode (thickness: 100 nm) was deposited on the HTL using a thermal evaporator. 

### 2.4. Characterization and Measurement

The composition, crystallographic structure, and optical transmittance of the TIO films were investigated via energy-dispersive X-ray spectroscopy (EDS; EX-250, Horiba, Kyoto, Japan), X-ray diffraction (XRD; X’pert PRO, PANalytical, Malvern, Malvern Hills District, UK), and ultraviolet-visible-near-infrared (UV-Vis-NIR) spectroscopy (Cary 7000, Agilent, Santa Clara, CA, USA), respectively. Furthermore, the work function and VBM of the films were determined by means of ultraviolet photoelectron spectrometry (UPS; AXIS Nova, Kratos, Wharfside, Manchester, UK). The electrical properties were measured using a Hall measurement system (HEK-3000, EGK, Pasadena, TX, USA) at room temperature. The photovoltaic properties were investigated using a source meter (Keithley 2450, Keithley Instruments, Cleveland, OH, USA) under simulated solar light generated by a solar simulator (Newport Oriel Solar AAA Class, 94023A). The AM 1.5 G sun light (100 mW cm^−2^) was calibrated using a standard Si reference cell (Newport, 91150V). In addition, the photocurrent density-voltage (*J-V*) curves were measured at a scan rate of 100 mV s^−1^. The area of the shadow mask was 0.1 cm^2^.

## 3. Results and Discussion

**Figure 1:**
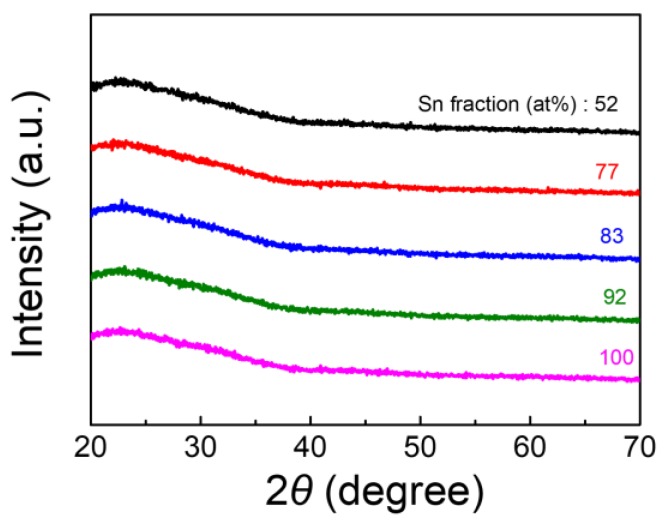
XRD patterns of TIO thin films with various Sn fractions.

For the EDS and XRD analyses, 250 nm-thick TIO thin films were deposited on a glass substrate at room temperature. By adjusting the target-substrate distances and gun powers, we successfully prepared various films with Sn fraction ([Sn]/([In] + [Sn])) of 52 at%, 77 at%, 83 at%, 92 at%, and 100 at%. These films are referred to as TIO-52, TIO-77, TIO-83, TIO-92, and TIO-100, hereafter. XRD patterns of the TIO thin films (see Figure 1) reveal that the films are amorphous, as evidenced by absence of any diffraction peaks for sequential lattice planes, especially at 2θ value of 30.5° and 26.6° corresponding to crystalline (222) plane of In_2_O_3_ and (110) plane of SnO_2_. The amorphous phase of the TIO films was also confirmed by means of Raman analysis (results excluded from this manuscript).

**Figure 2:**
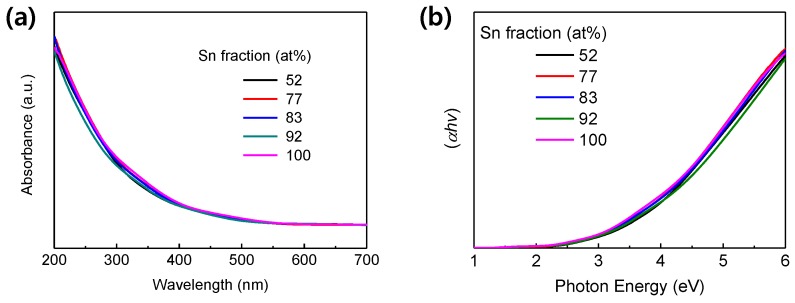
(**a**) UV-Vis absorbance and (**b**) Tauc plot of the ITO thin film with different Sn contents.

Figure 2a shows UV-Vis absorbance of the TIO films. 250 nm-thick TIO films were prepared on sapphire substrates having a wide band gap (~10 eV), because ITO or glass substrates absorb near-UV light near to the band edge wavelength of TIO, and this absorption may prevent the acquisition of accurate absorption edges. The samples exhibit similar absorbance levels and absorption edges. The optical band gap energies of the TIO films were determined from the Tauc plots with r = 1 (see Figure 2b), which were converted from the absorbance spectra using the Tauc equation, αhν = A(hν-Eg)^r^; α, hν, A, and the exponent r denote the absorption coefficient, photon energy, proportionality constant, and the nature of the transition, respectively. In this work, non-linear curves were obtained for the plots with r = 1/2 (for direct transition) and r = 2 (for indirect transition), although crystalline In_2_O_3_ and crystalline SnO_2_ are direct transition materials [42]. In contrast, linear curves were obtained with r=1, which relation has been reported from materials having a very narrow range of localized states [43]. The optical band gap energy increases slightly (3.49, 3.52, 3.57, 3.62, and 3.65 eV for TIO-52, 77, 83, 92, and 100, respectively) with increasing Sn fraction. The band gap energy of TIO-100 (i.e., amorphous SnO_2_) determined in this study is very similar to that of crystalline SnO_2_ (~3.7 eV) reported in the literature [44].

**Figure 3:**
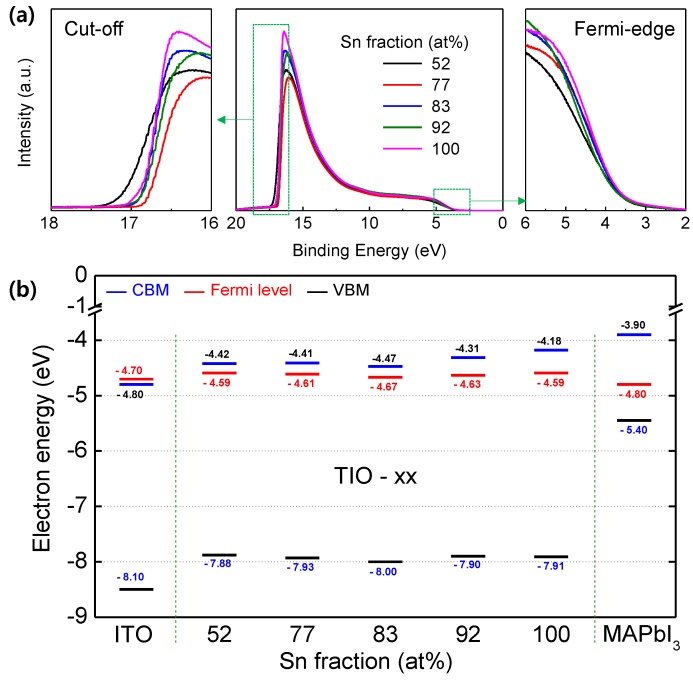
(**a**) UPS spectra of TIO films with different Sn contents. The middle panel, left panel, and right panel show the entire range, cut-off edge region, and Fermi-edge region, respectively. (**b**) CBM, VBM, and Fermi levels of the TIO films. The levels corresponding to ITO and MAPbI_3_ are taken from previous studies [45,46].

In order to investigate the energy band structure of TIO, the UPS spectrum was collected, as shown in Figure 3a. The Fermi level (E_F_) was determined through linear extrapolation of the leading edge at the cut-off region (E_F_ = E_cut-off_ − 21.2 eV). The VBM was determined via linear extrapolation of the leading edge at the Fermi edge region (E_VBM_ = E_F_ − E_fermi edge_). The CBM was obtained by combining the VBM and the optical band gap energy (E_CBM_ = E_VBM_ + E_g_). Figure 3b shows the energy levels of the TIO films. These levels exhibit no distinct dependence on the Sn fraction. The CBM of each sample is higher than that of ITO and lower than that of MAPbI_3_, and is therefore favorable for electron transfer from MAPbI_3_ to ITO through TIO. In addition, good blocking of the holes from MAPbI_3_ is expected, because each VBM of the TIO is positioned at lower energy levels than that of MAPbI_3_. Another notable point is that the energy difference between the CBM and the Fermi level increases (0.17, 0.20, 0.20, 0.32, and 0.41 eV for TIO-52, 77, 83, 92, and 100, respectively) with increasing Sn fraction. This implies that the electrical conductivity would decrease with increasing Sn fraction, owing to a reduction in the carrier concentration.

**Figure 4:**
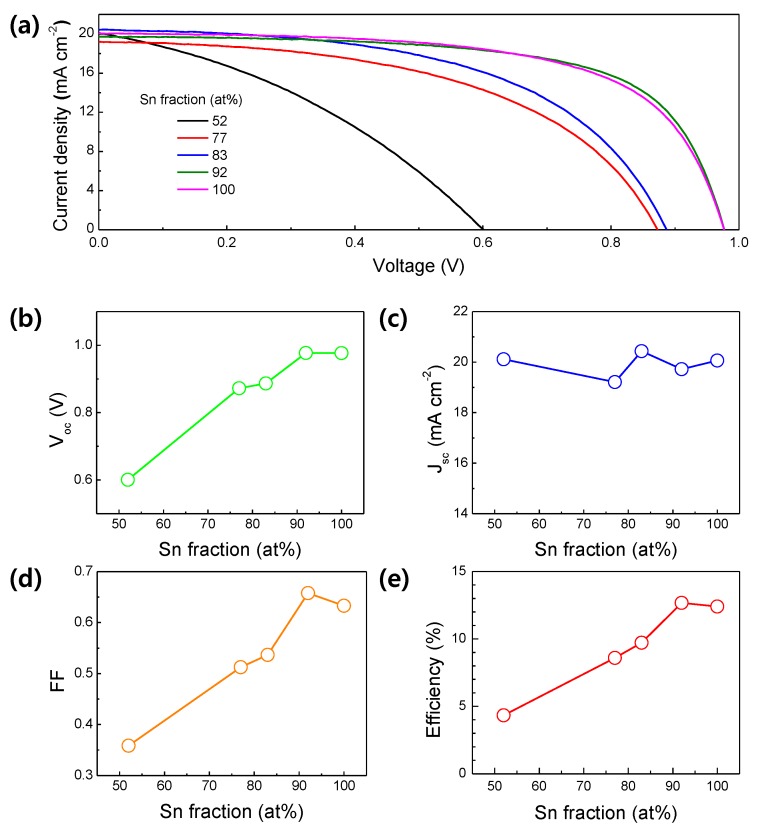
(**a**) J-V curves of the TIO-ETL-based PSCs with different Sn fractions. The curves were collected under the following conditions: solar simulated light of AM 1.5G, 100 mV cm^−2^, scan rate (reverse) of 100 mV s^−1^, active area of 0.1 cm^2^. The photovoltaic parameters extracted from the curves; (**b**) V_oc_, (**c**) J_sc_, (**d**) FF, and (**e**) PCE.

Using the TIO thin films as the ETL, regular planar-type PSC devices were fabricated. The PSCs have a glass/ITO/TIO-ETL/MAPbI_3_/HTL/Au structure. The thickness of the TIO-ETL (i.e., 40 nm) was controlled by adjusting the sputtering time. The ETL, HTL, and Au layers were deposited at room temperature and, hence, the highest processing temperature is 130 °C which is associated with the perovskite LAL coating. Figure 4a shows the J-V curves of the TIO-ETL-based PSCs, measured under 1 SUN (1.5 G) condition. The plots in Figure 4b–e show the photovoltaic parameters, such as the open circuit voltage (V_oc_), short circuit current density (J_sc_), fill factor (FF), and power conversion efficiency (PCE), of the PSCs. The results show that the V_oc_ and FF increase significantly (from 0.6 V to 0.98 V and 0.36 to 0.66, respectively) with increasing Sn fraction. However, the J_sc_ remains almost a similar level, c.a. 20 mA/cm^2^ with a small deviation less than 10%, regardless of composition. Consequently, the PCE increases approximately three-fold, from 4.33% (TIO-52) to 12.67% (TIO-92), owing mainly to the enhanced V_oc_ and FF. A comparison of the J-V curve shapes reveals that the photocurrent density of the ITO-52-based PSC decreases steeply with increasing voltage. A steep slope in the low-voltage regions combined with a high J_sc_ and a low V_oc_ occurs when the shunt resistance of the diode element is small. In contrast, with increasing Sn fraction, the curves transfer to more diode-like characteristics, i.e., a gentle slope up to ~0.8 V and a steep slope at voltages above this value. High Sn fractions yield curves that approach the ideal J-V curve, indicating increased shunt resistance. The interfacial charge-carrier dynamics of MAPbI_3_ and TIO-ETL were evaluated by impedance analysis. The Nyquist plots demonstrate substantial difference in the radius arc of semicircle (Figure S1, Supplementary Material). The semicircle in the Nyquist spectra at lower frequency is mainly ascribed to the recombination resistance (R_rec_) [18]. Clearly, from the EIS plot of Figure S1, the R_rec_ of TIO-ETL PSCs increases monotonically as the fraction of Sn increases. Therefore, adding Sn increases the shunt resistance, leading to suppression of the interfacial charge recombination. On the other hand, the highest FF (0.66) is obtained for the TIO-92 PSC, rather than for the TIO-100 (i.e., In-free SnO_2_; FF = 0.63) PSC. The decrease in the FF results in a reduction (from 12.67% to 12.40%) in PCE. Compared with the slope of the TIO-92 PSC J-V curve, the slope of the TIO-100 PSC curve is gentler at voltage values over 0.8 V, but is similar at lower voltage levels. The lower FF of the TIO-100 PSC (compared with that of the TIO-92 PSC) can therefore be attributed to an increase in the series resistance of the TIO-100 PSC.

**Figure 5:**
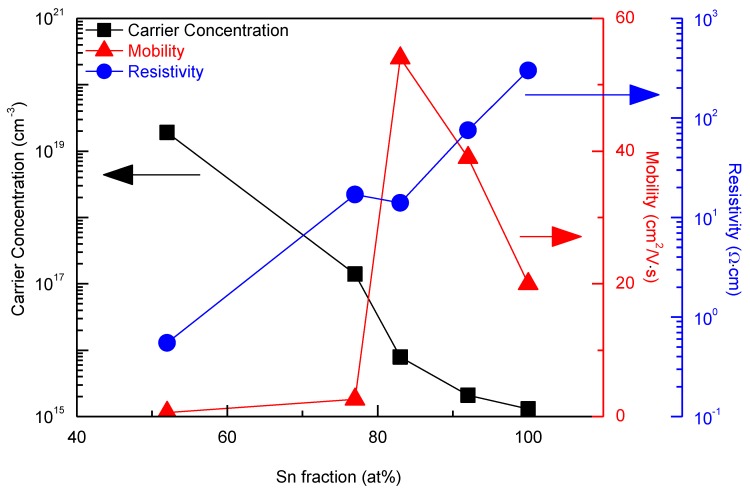
Carrier concentration, mobility, and resistivity of the TIO thin films.

In general, crystalline commercial ITO (Sn fraction: ~10 at%) thin films used for TCO are characterized by a carrier concentration of ~10^21^/cm^3^. Many studies have considered the effect of low Sn content (Sn fraction: ~10 at%) on the properties of ITO films [47,48,49,50]. However, the effect of high Sn fraction, especially in the case of the amorphous phase, remains unexplored. In order to understand the changes in shunt and series resistances of the PSCs with varying the Sn fraction in the TIO ETL, the electrical properties such as carrier concentration, mobility and resistivity of the TIO films were investigated by Hall effect measurements with Van der Pauw configurations. Figure 5 shows that the carrier concentration of TIO decreases significantly (i.e., by four orders of magnitude) from 1.9 × 10^19^/cm^3^ to 1.3 × 10^15^/cm^3^, as the Sn fraction increases. This trend is consistent with the results shown in Figure 3b that the energy difference between the CBM and Fermi level increases as the Sn fraction increases. It is reasonable that the high carrier concentration leads to the shallow Fermi level, and vice versa. The mobility also changes considerably from 0.61 cm^2^/V-s to 54 cm^2^/V-s, but the difference is relatively small compared with that in the carrier concentration. This leads eventually to a significant increase (up to three orders of magnitude) in the resistivity of TIO. Therefore, by combining the resistivity of the TIO-ETL and the photovoltaic characteristics of the TIO-based PSCs, the shapes of the J-V curves could be explained. The very low resistivity (with very high electron density) of TIO-52 would facilitate electron back transfer from the TCO to perovskite LAL, leading to a decrease in the shunt resistance of the device. With increasing Sn fraction, the resistivity of TIO ETL increases, resulting in improved blocking of the back electron transfer. However, at a Sn fraction of 100 at%, the resistivity becomes excessively high (as in the case of an insulator) and the mobility decreases to levels lower than those of the TIO-83 and 92. This would lead to a slight increase in the series resistance of the device. Therefore, the effect of the shunt resistance and the effect of series resistances may compete, leading to the optimal Sn fraction of ~92 at%.

**Figure 6:**
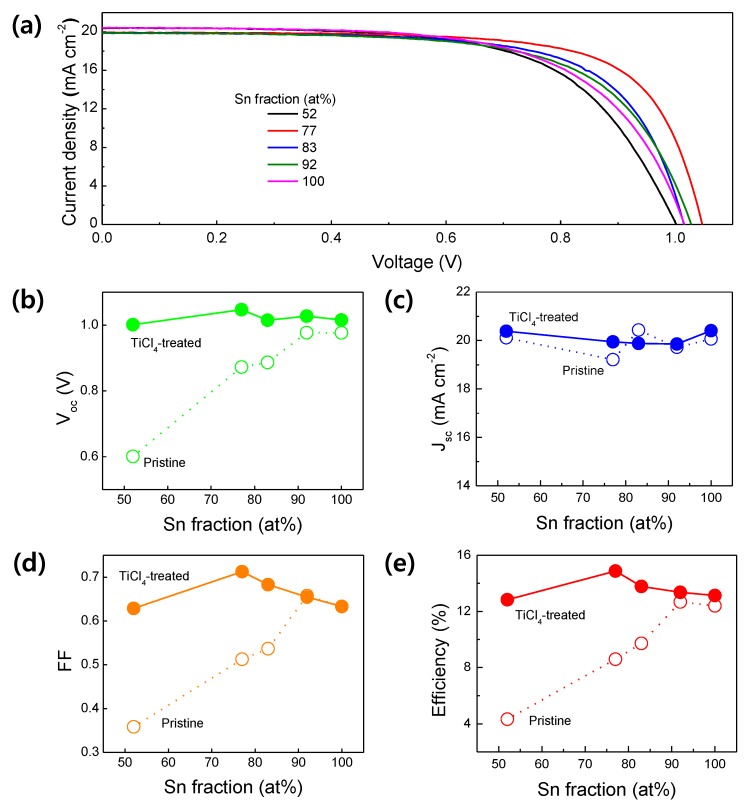
(**a**) J-V curves of the TiCl_4_-treated TIO-ETL-based PSCs with different Sn fractions. The curves were collected under the following conditions: solar simulated light of AM 1.5G, 100 mV cm^−2^, scan rate (reverse) of 100 mV s^−1^, active area of 0.1 cm^2^. The photovoltaic parameters extracted from the curves: (**b**) V_oc_, (**c**) J_sc_, (**d**) FF, and (**e**) PCE. For comparison, the parameters of the untreated TIO-ETL-based PSCs (from Figure 5; indicated as pristine here) are inserted in (**b**–**e**).

The best device based on the TIO ETLs exhibits still low FF and V_oc_, compared to common PSCs based on solution processed TiO_2_ or SnO_2_ ETLs. The low FF and V_oc_ are attributed to the low shunt resistance of the TIO-based PSCs. To further increase the shunt resistance, we applied a TiCl_4_ treatment to the TIO ETLs. Figure 6a shows the J-V curves of the TiCl_4_-treated TIO-PSCs. The corresponding photovoltaic parameters are shown in Figure 6b–e. Interestingly, the TiCl_4_ treatment increases each photovoltaic parameter of the PSCs, over the entire Sn fraction range. The corresponding J-V curves are closer to that of the ideal diode, as evidenced by the flatter slope in the low-voltage region and steeper slope in the vicinity of V_oc_, compared with the J-V curves of the pristine TIO-PSCs shown in Figure 4a. In general, the TiCl_4_ treatment yields an additional very thin titanium oxide layer that shields pin holes and cracks on the ETL. This shielding prevents direct contact between the TCO and the LAL, thus reducing the charge recombination [51,52,53,54,55,56,57]. The recombination resistance of a solar cell is correlated with the shunt resistance. Therefore, the improved performances of the TiCl_4_-treated TIO-based PSCs are attributed to the improved diode nature resulting from the increased shunt resistance. Owing to this increase, the TiCl_4_ treatment leads to a significant increase in the V_oc_ and FF of the devices (Figure 6b,d), especially those with low Sn fractions. Interestingly, the highest V_oc_ and FF of the TiCl_4_-treated device are obtained from the TIO-77 PSCs, while those of the pristine devices are obtained from the TIO-92 PSCs. This difference can be understood by noting that all the samples have similar slopes (i.e., similar shunt resistance) in the low-voltage region, but very different slopes (i.e., diverse series resistance) in regions near V_oc_. Considering the gradually decreasing FF with increasing the Sn fraction at >77at% mainly due to becoming gentler J-V slope at the vicinity of V_oc_, we attribute the deteriorated V_oc_ and FF to increased series resistance of the devices. Therefore, it can be concluded that the series resistance rather than the shunt resistance becomes more influential factor when the TIO is TiCl_4_-treated. Owing to this strengthened effect, the optimal Sn fraction is shifted to 77 at% of which resistivity is much lower than 92 at%. Eventually, the trend describing the PCE as a function of the Sn fraction becomes similar to that of FF, because the V_oc_ and the J_sc_ change modestly, compared with the FF. 

The TiCl_4_-treated TIO-PSC exhibits the best PCE of 14.88% with 19.95 mA/cm^2^ of J_sc_, 1.05 V of V_oc_ and 0.713 of FF at 77at% of Sn fraction, and the pristine TIO-PSC exhibits the best PCE of 12.67% with 19.72 mA/cm^2^ of J_sc_, 0.977 V of V_oc_ and 0.658 of FF at 92 at% of Sn fraction, as summarized in Table S1 (Supplementary Material). The J-V hysteresis of several devices was examined, as shown in Figure S2 (Supplementary Material), by measuring the J-V along the opposite voltage sweep directions (reverse and forward scanning). Regardless of the TiCl_4_ treatment, the TIO-PSCs exhibit small J-V hysteresis (HF ranging 0.16–0.25). For the last, the photovoltaic performance of the TIO-ETL-based PSC was tracked during 360 hr to examine the long term stability of the device, as shown in Figure S3 (Supplementary Material). After 360 h of storage (at room temperature, 20% of relative humidity), the PCE of the TIO-ETL-based PSC maintained 98% of its initial value, whereas the PCE of the commercial SnO_2_ ETL-based PSC degraded to 77%.

## 4. Conclusions

In summary, amorphous TIO thin films with a Sn fraction of 52 at%–100 at% were grown (via RF magnetron co-sputtering) on ITO substrates at room temperature, for application to the ETLs of PSCs. The optical band gap of the films slightly increases from 3.49 eV to 3.65 eV with increasing (from 52 at% to 100 at%) Sn fraction. The CBMs of the films are located between those of ITO and MAPbI_3_, a configuration that is desired for electron transport in PSCs. The electrical resistivity increases considerably (up to three orders of magnitude) with increasing Sn fraction, owing mainly to a fourth order decrease in the electron concentration. This is consistent with the widening of the energy difference between the CBM and the Fermi level with increasing Sn fraction. The PSCs based on the TIO ETLs are fabricated. Due to the competition between the shunt and series resistances, the optimal photovoltaic performance is obtained at a Sn fraction of 92 at%. The TiCl_4_ treatment of the TIO ETL results in increased shunt resistance, leading to improved photovoltaic performance over the entire range of Sn fractions. Furthermore, the optimal PSC performance (J_sc_: 19.95 mA/cm^2^, V_oc_: 1.05 V, FF: 0.713, PCE: 14.88%) is realized at a Sn fraction of 77 at%. The TIO-based PSCs exhibit small J-V hysteresis, and good long-term stability. Our result demonstrates that the tin-indium-oxide film is a promising candidate for use as an ETL for PSCs. The TIO ETL can be continuously grown on a pre-sputtered ITO in a chamber, as ITO and TIO are composed of identical elements (In, Sn and O), which would help to reduce production time and costs. Moreover, the PCE can be further improved by developing a solution process for TIO thin films.

## Supplementary Materials

The following are available online at https://www.mdpi.com/1996-1944/13/1/32/s1, Figure S1: The electrochemical Nyquist plots of TIO-ETL with varied Sn (at%) fractions, Figure S2: J-V curve of the optimal PSCs based on the pristine TIO-ETL and the TiCl4-treated TIO ETL, Figure S3: Photovoltaic properties tracked for a long-term (15 days) to compare the stability of the PSCs based on our TIO (Sn-83at%) ETL with that of a commercial SnO_2_ ETL, Table S1: Photovoltaic parameters of the optimal PSCs.
