# Supplementary material for: Room-Temperature-Processed Amorphous Sn-In-O Electron Transport Layer for Perovskite Solar Cells
Source: Materials (Basel). 2019 Dec 19;13(1):32. doi: 10.3390/ma13010032 (PMC6981739; doi:10.3390/ma13010032)
Supplement: Supplementary file 1 [file materials-13-00032-s001.pdf]

materials

Supplementary Material

Room-Temperature-Processed Amorphous Sn-In-O Electron Transport Layer for Perovskite Solar Cells

Seungtae Baek [1], Jeong Woo Han [1], Devthade Vidyasagar [1], Hanbyeol Cho [1], Hwi-Heon HA [1], Dong Hoe Kim [2], Young-Woo Heo [1,*] and Sangwook Lee [1,*]

[1] School of Materials Science and Engineering, Kyungpook National University, Daegu 41566, Republic of Korea; en5840@knu.ac.kr (S.B.), wjddn0820@knu.ac.kr (J.W.H.), vidyasagar.devtade@gmail.com (D.V.), jhb1005@gmail.com (H.C.), gkgnlgjs5@naver.com (H.-H.H.).

[2] Department of Nanotechnology and Advanced Materials Engineering, Sejong University, Gwangjin-gu, Seoul 05006, Republic of Korea; donghoe.k@sejong.ac.kr

* Correspondence: ywheo@knu.ac.kr (Y.-W.H.), wook2@knu.ac.kr (S.L.); Tel.: +82-53-950-5632

Received: 4 November 2019; Accepted: 16 December 2019; Published: date

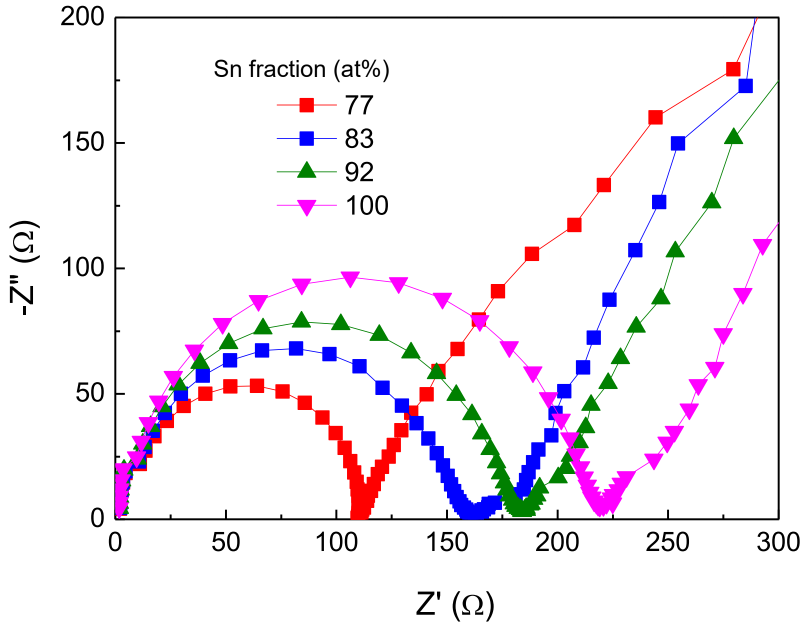

Figure S1. The Nyquist plots of TIO-ETL cells with varied Sn (at%) fractions (under 1 SUN illumination without applied bias).

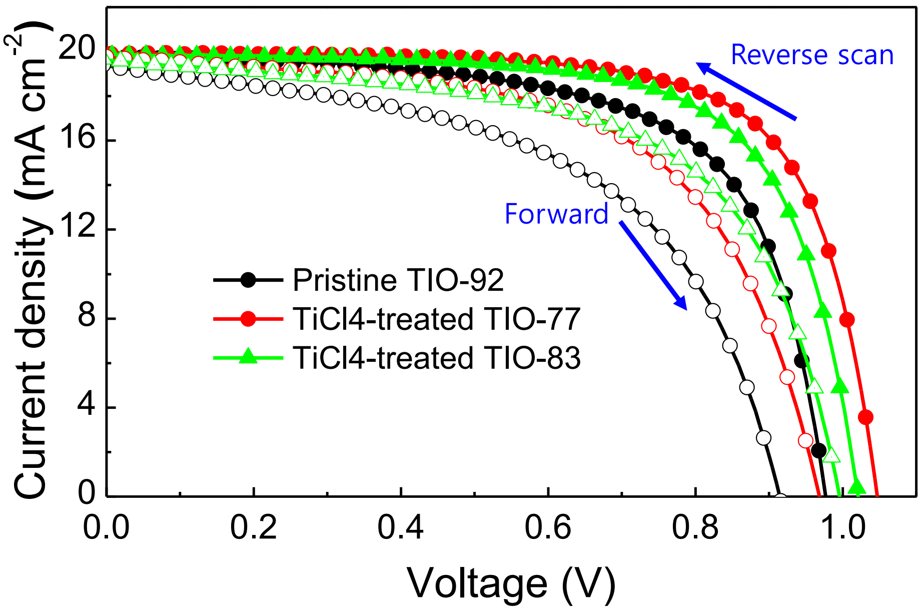

Figure S2. J-V curves of the optimal PSCs based on the pristine TIO-ETL (Sn fraction: 92 at%) and the $TiCl_4$-treated TIO ETL (Sn fraction: 77 at%). The $TiCl_4$-treated TIO-83, with the smallest hysteresis, is also presented.

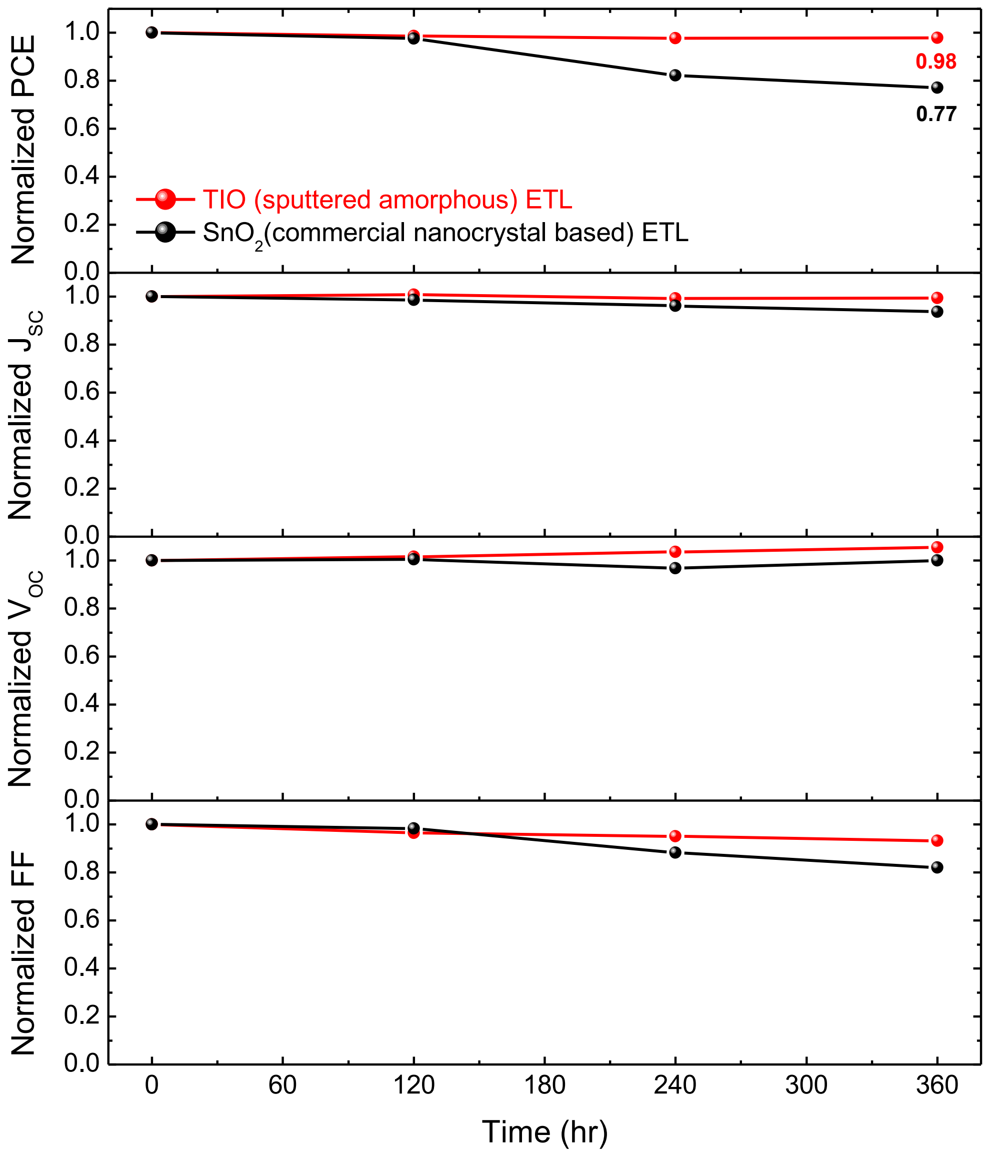

Figure S3. Photovoltaic properties tracked for the long-term (15 days) to compare the stability of the PSCs (with triple-cation perovskite LAL) based on the TIO ETL and a commercial SnO_2 ETL.

Table S1. Photovoltaic parameters of the optimal PSCs based on the pristine TIO-ETL and the $TiCl_4$-treated TIO ETL. The $TiCl_4$-treated TIO-83, which exhibits the smallest hysteresis, is also shown.

TIO-ETL	J_{sc} [mA·cm^{-2}]	V_{oc} [V]	*FF*	*PCE* [%]	*HF**
Pristine (92 at%)	19.72	0.98	0.66	12.67	0.25
$TiCl_4$-treated (77 at%)	19.95	1.05	0.71	14.88	0.24
$TiCl_4$-treated (83 at%)	19.86	1.02	0.69	14.07	0.16

*HF: hysteresis factor (= 1 – $PCE_{forward}/PCE_{reverse}$).
